# Nonconsumptive effects of hunting on a nontarget game bird

**DOI:** 10.1002/ece3.5479

**Published:** 2019-07-30

**Authors:** Jessica L. Mohlman, Rachel R. Gardner, I. B. Parnell, Nathan G. Wilhite, James A. Martin

**Affiliations:** ^1^ D. B. Warnell School of Forestry and Natural Resources University of Georgia Athens GA USA; ^2^ Wildlife Resources Division Georgia Department of Natural Resources Thomson GA USA; ^3^ Savannah River Ecology Lab University of Georgia Athens GA USA

**Keywords:** antipredator behavior, before‐after‐control‐impact design, hunting, nonconsumptive effects, Northern Bobwhite, protean behavior, risky space, threat sensitivity

## Abstract

1. Human hunting activity and disturbance can significantly impact prey species through both consumptive and nonconsumptive effects. The nonconsumptive effects of rabbit hunting on Northern Bobwhite (*Colinus virginianus*; hereafter, bobwhite) are currently unknown. Increased perceived risk of predation by bobwhite during rabbit hunting events may elicit antipredator responses among bobwhite that impact fitness via changes in behavior that ultimately impact population growth.
2. We estimated the nonconsumptive effects of rabbit hunting on bobwhite behavior using telemetry across varying rabbit hunting intensities. Movements were analyzed using Bayesian hierarchical modeling with a before‐after‐control‐impact (BACI) design to determine the effect of rabbit hunting on bobwhite.
3. We observed an overall reduction in bobwhite movement in the presence of rabbit hunting, with a 38% (Posterior Overlap = 0.01) increase in bobwhite step length in the absence of rabbit hunting. We also observed bobwhite maintaining closer proximity to hardwood and escape cover under high rabbit hunting intensity, with a 59% (Posterior Overlap = 0.03) increase in distance from hardwood and a 28% (Posterior Overlap = 0.14) increase in distance from escape cover when rabbit hunting was removed.
4. *Synthesis and applications*. Heightened antipredator behavior through decreased movement may assist with bobwhite predator avoidance. However, decreased movement and increased use of poor habitats may also have negative effects as a result of reduced foraging time or increased susceptibility to other predators. Future research should attempt to quantify the effect of decreased movement on bobwhite fitness through the evaluation of foraging time and survival in order to continue to improve management efforts for the species.

## 1 INTRODUCTION

Understanding predator–prey dynamics and the complexity of community ecology is critical for effective management and wildlife conservation. Anthropogenic disturbances can have profound effects on wildlife by eliciting fear and altering behavior (Gaynor, Hojnowski, Carter, & Brashares, 2018). This fear is often evoked indirectly through auditory and visual anthropogenic cues (e.g., vehicles and construction; Smith et al., 2017). It has been recognized that such human‐driven disturbances can have cascading effects across food webs (Smith et al., 2017; Smith, Wang, & Wilmers, 2015). Within many systems, human hunting activity can exert a greater threat to prey than natural predators (Allendorf & Hard, 2009; Creel & Christianson, 2008; Darimont et al., 2009). Additionally, human hunters are unique apex predators as they are bound by regulations and bias that often dictate prey habitat use and spatial patterns (Asmyhr, Willebrand, & Hörnell‐Willebrand, 2013; Cromsigt et al., 2013; Lone et al., 2014; Stillfried, Belant, Svoboda, Beyer, & Kramer‐Schadt, 2015). Hunting disturbance is known to cause antipredator behavior in prey as a way to mitigate the risk of predation (Lima & Bednekoff, 1999). Prey species attempt to mitigate these risks through behaviors such as altered habitat use and increased vigilance (Creel, Winnie, Maxwell, Hamlin, & Creel, 2005; Clinchy et al., 2016; Embar, Raveh, Burns, & Kotler, 2014; Frid & Dill, 2002; Lima & Dill, 1990). However, antipredator behavior can have fitness costs in terms of reduced foraging effort, greater net energy loss, and increased vulnerability to other predators (Lima & Bednekoff, 1999; Morrison, 1999; Downes, 2001; Eklov & Van Kooten, 2001; Creel, 2018). Though ecological theory addresses both direct and indirect and lethal and nonconsumptive effects of predation, wildlife management often focuses specifically on lethal consumptive effects (Preisser, Orrock, & Schmitz, 2007). However, nonconsumptive effects of hunting can at times be stronger than lethal effects through altered prey behavior (Clinchy et al., 2016; Frid & Dill, 2002; Lima & Bednekoff, 1999). The threat sensitivity hypothesis states that “individuals will trade‐off predator avoidance against other activities by altering their avoidance responses in a manner that reflects the magnitude of the predatory threat” (Helfman, 1989). Prey species' perception of predation risk and subsequent decisions are not necessarily a result of direct predator confrontation, but often the result of indirect landscape cues (Schmidt & Kuijper, 2015). With regards to hunting, prey are exposed to numerous indirect cues such as the auditory cues of hunting dogs and firearms and increased landscape disturbance within these areas (Ciuti et al., 2012; Cromsigt et al., 2013). Preys' response to their perception of risk alone may be powerful enough to affect wildlife population dynamics (Zanette, White, Allen, & Clinchy, 2011). Therefore, it is important to have a thorough understanding of nonconsumptive hunting impacts on prey species (Kotler & Holt, 1989).

Nonconsumptive impacts of hunting may be particularly important for understanding the fitness of nontarget game and nongame wildlife. Many game and nongame species are managed in landscapes that often include other game species. As a result, hunting and management activities targeted at one species may have negative nonconsumptive effects on other species. This effect may be amplified if the nontarget prey is also a game species and cannot distinguish whether it is the intended target of predation. Prey response to the threat of predation may be directly correlated with its perception of the encounter (Blumstein, 2003; Edgar, Paul, & Nicol, 2013). While there has been a substantial increase in the study of direct and indirect effects of hunting on prey (Gosselin, Zedrosser, Swenson, & Pelletier, 2014; Lone, Loe, Meisingset, Stamnes, & Mysterud, 2015; McGrath, Terhune, & Martin, 2018; Stillfried et al., 2015), the nonconsumptive effects of hunting on nontarget prey species are understudied.

Northern Bobwhite (*Colinus virginianus*; hereafter, bobwhite) are a commonly hunted species within the United States and share hunting seasons and habitats with many small game species, such as Eastern cottontails (*Sylvilagus floridanus*; hereafter, rabbit). Bobwhite have experienced considerable population declines since the 1920s (Brennan, 1991; Hernández, Brennan, DeMaso, Sands, & Wester, 2013; Stoddard, 1931). Broad‐scale changes in land use including modern agricultural and forestry practices have resulted in habitat loss that is the primary cause of this decline (Brennan, 1991; Guthery, Peterson, & George, 2000; Williams, Guthery, Applegate, & Peterson, 2004). Bobwhite popularity as a game species, in conjunction with their declines, has made the species a conservation priority and prompted their designation as a flagship species for many upland and grassland bird communities (Brennan & Kuvlesky, 2005; Crosby, Elmore, Leslie, & Will, 2015).

The effect of rabbit hunting on bobwhite antipredator behavior is currently unknown but is a management concern due to the fact that both species coincide in the same habitats and have concurrent hunting seasons. Increased perceived risk of predation by bobwhite during rabbit hunting events may elicit antipredator behavior or alterations to other behavior that, in turn, can affect demography and factors related to hunt quality (e.g., encounter rates). We hypothesized that bobwhite respond to cues during rabbit hunting as a direct threat and mitigate the perceived threat following the predictions of the threat sensitivity hypothesis (Helfman, 1989). We tested this hypothesis by subjecting bobwhites to varying levels of rabbit hunting and predicted that behavior would change across this gradient. We relied on expert knowledge and the literature on bobwhite (McGrath et al., 2018) to make more specific predictions under this theoretical framework to help uncover possible behavioral patterns and strategies. Specifically, we predicted that bobwhites may mitigate risk by reducing movement (i.e., shorter step length and contracted space use) under greater rabbit hunting or increase movement because of more frequent threat encounters. Bobwhite may use Protean behavior which involves more erratic movement patterns in an effort to avoid predation (Jones, Jackson, & Ruxton, 2011), which would be reflected in increased path tortuosity of movement. Moreover, we hypothesized bobwhite would follow the risky space hypothesis (Creel, Winnie, Christianson, & Liley, 2008; Cresswell & Quinn, 2013; Laundré, Hernández, & Altendorf, 2001) by maintaining closer proximity to escape cover (e.g., scrub/shrubs) and hardwoods where there is less hunting pressure but also poorer quality resources (Lima, 1993; Lohr, Collins, Castelli, & Williams, 2010; Wilhite, 2019).

## 2 MATERIALS AND METHODS

### 2.1 Study site

The research was conducted on Di‐Lane Wildlife Management Area (WMA), located in Waynesboro, Georgia. The study area encompassed roughly 3,278 ha with ~2,023 ha managed for bobwhite by the Georgia Department of Natural Resources since 1993. Prior to its designation as a public WMA, the property was a private quail plantation and agricultural farm. Habitat management for bobwhite also benefits rabbits, leading to robust populations of both species on the site.

Bobwhite hunting season occurs during the month of December on the property, with two additional hunts the first week of February (10 total hunts). All bobwhite hunts on the property are quota hunts and occur every Wednesday and Saturday during the season. Quota hunts restrict the amount of bobwhite hunters who can participate on each day of the season (maximum of 24 hunters per day). Rabbit hunting falls under the category of small game species on the property and is not on a quota hunt system—the number of rabbit hunters is not restricted during the open season. Rabbit season takes place early November through February. During bobwhite hunting season, rabbits may be hunted any day of the week except concurrently with bobwhite hunting. Both species are hunted on foot within similar habitats and with the use of hunting dogs.

### 2.2 Experimental design

**Figure 1:**
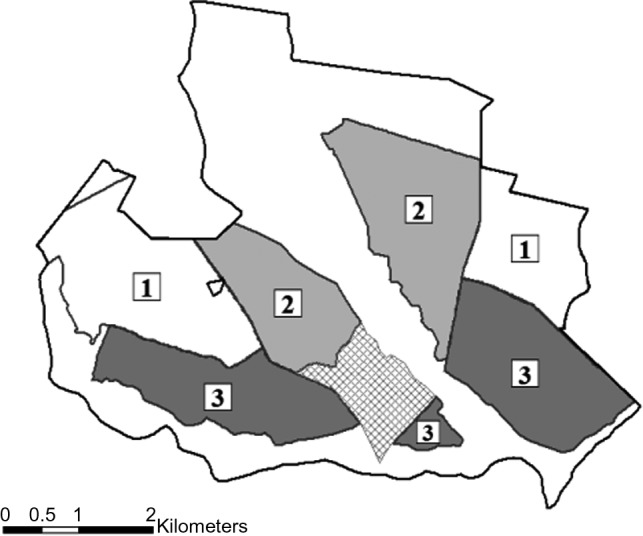
Rabbit hunting treatments on Di‐Lane Wildlife Management Area, located in Waynesboro, GA. Treatment 1 (No Rabbit Hunting) indicates 0 days of rabbit hunting per week, Treatment 2 (Reduced) indicates 3 rabbit hunting days per week, and Treatment 3 (Reference) indicates 5 rabbit hunting days per week. The gridded area specifies the safety zone in which no hunting can occur

The study involved a before‐after‐control‐impact (BACI) design (Stewart‐Oaten, Murdoch, & Parker, 1986; Underwood, 1994). During the before period of 2016–2017, rabbit hunting opportunities were homogenous across the study site with hunting occurring 5 days per week. This was followed by an after period during 2017–2018 in which rabbit hunting treatments were assigned across the study site in a randomized complete block design (i.e., three levels of rabbit hunting and two spatial blocks). The treatments included 0 days of hunting per week (“No Rabbit Hunting”), 3 days of hunting per week (“Reduced Hunting”), and 5 days of hunting per week (“Reference”), with replicates of each treatment (Figure 1). The BACI design allows for treatment impacts to be distinguished from background time effects shared by all the replicates and background differences between the treatment and reference sites (Popescu, de Valpine, Tempel, & Peery, 2012).

### 2.3 Field methods

#### 2.3.1 Bobwhite trapping and processing

Walk‐in funnel traps baited with sorghum were used to capture bobwhite (Stoddard, 1931). Trapping occurred both years from early October to early November, prior to rabbit hunting season. Traps were uniformly spaced 250–300 m apart across the property within dense cover. All unique individuals captured were weighed, sexed, aged, and given unique number leg bands (National Band & Tag Company) when processed. Individuals captured that weighed ≥130 g were fitted with Very High Frequency (VHF) radio tags from Holohil Systems and American Wildlife Enterprises. During fall and winter, bobwhite congregate in social groups called coveys (DeMaso, Guthery, Spears, & Rice, 1992; Williams, Lutz, & Applegate, 2003). Caution was taken to not deploy more than 6 radio tags per covey to allow for uniform dispersion of tags across the study site and rabbit hunting treatments. Within a given trapping session, between 150 and 170 radio tags were deployed. Each tag had an estimated battery life of 10–12 months and was equipped with mortality signals. If tagged individuals remained inactive for >12 hr, the signals switched to a rapid mortality beep indicating the individual was deceased.

#### 2.3.2 Radio telemetry of bobwhite

Bobwhite coveys were monitored to capture fine‐scale movement patterns using radio telemetry. Coveys were tracked from approximately 30 min prior to sunrise to approximately 30 min after sunset to capture movement during active periods. Locations were determined via homing telemetry (Amelon, Dalton, Millspaugh, & Wolf, 2009; White & Garrott, 1990) at 30‐min intervals. Approximate locations of coveys were taken roughly 20–30 m from the observer to minimize disturbance, with an estimated error rate of 12 m. Global positioning systems (GPS) were used to note observer locations, and compasses were used to note the azimuth to the covey. Intensive telemetry occurred once on all coveys fitted with radio tags during the scope of the study, with individuals being tracked on either a rabbit hunting or bobwhite hunting day throughout the duration of both hunting seasons. Coveys were selected for tracking through random number generation to reduce observer bias.

### 2.4 Statistical analysis

Statistical analyses were performed using R (R Core Team, 2017). Bobwhite movement data from November 2016 to February 2017 were analyzed for the before‐time period, and data from November 2017 to February 2018 were analyzed for the after‐time period to encompass the full duration of the rabbit hunting seasons. We assumed coveys remained in their respective rabbit hunting treatments for the duration of the treatment year due to the size of the treatments (range: 218.65–383.45 ha) and the decreased dispersal of bobwhite during the overwintering months (Townsend et al., 2003).

### 2.5 Trajectory and habitat use analysis

For trajectory analysis, metrics of interest such as step length, straightness, trajectory distance, and straight‐line trajectory distance were calculated with the package “trajr” (McLean & Skowron Volponi, 2018). Step length denotes the distance that an individual moved between successive observed locations. Path straightness indicates whether path trajectories were linear or tortuous on a scale of 0–1 with perfectly straight paths represented by 1 (McLean & Skowron Volponi, 2018). Trajectory distance is the cumulative observed movement distance in meters (McLean & Skowron Volponi, 2018). Straight‐line trajectory distance indicates the distance from the start to the end of a trajectory path and not the cumulative distance of movement throughout a track (McLean & Skowron Volponi, 2018). Habitat analyses were conducted using Sentinel‐2 imagery data generated by the European Space Agency (ESA) and provided by the Geological Survey (USGS) at a resolution of 10 m. Supervised classification was conducted in Google Earth Engine (Gorelick et al., 2017) by generating training polygons of the desired land cover types (e.g., hardwood and scrub/shrub) in ArcGIS (ESRI, 2017). An assessment of map accuracy was conducted by generating 100 equally distributed random points across each land cover type of interest and generating an error matrix compared to ground‐truthed values. Map accuracy was determined to be 100% for hardwoods and 77.41% for scrub/shrub vegetation. To determine bobwhite use of hardwood and scrub/shrub, the Euclidean distance from each individual location to the nearest example of that habitat feature was calculated.

### 2.6 Bayesian hierarchical modeling and BACI ratio

We used a Bayesian hierarchical modeling approach within the jagsUI package (Kellner, 2018) of R (R Core Team, 2017) to estimate the effects of the treatment and reference groups before and after the implementation of rabbit hunting treatments using the model described below. We assumed normal distributions for the random effects with a mean of 0 and vague gamma‐distributed precision terms (1/variance). We used vague normal priors for the fixed effects with a mean of 0 with small precision (0.001). The model was parameterized using the “effects” parameterization where the fixed effects represented the difference from the reference treatment in the before‐time period. The general model is as follows

$$\mu_{i}=\beta_{\text{Ref}\;\text{Before}}+\beta_{\text{Norab}}\times X_{\text{Norab}}+\beta_{\text{Red}}\times X_{\text{Red}}+\beta_{\text{Norab}\;\times \;\text{After}}\times X_{\text{Norab}\;\times \;\text{After}}+\beta_{\text{Red}\;\times \;\text{After}}\times X_{\text{Red}\;\times \;\text{After}}+\sigma_{S}$$

where $M_{i}∼\text{Norm}\left(\mu_{i},\;\tau \right)$ is the model likelihood and

$$\tau ∼\text{Gamma}\left(0.1,\;0.1\right);$$

$$\beta_{k}∼\text{Norm}\left(\mu_{k},\;\tau_{k}\right);$$

$$\mu_{k}∼\text{Norm}\left(0,\;0.001\right);$$

$$\tau_{k}∼\text{Gamma}\left(0.1,\;0.1\right);$$

$$\sigma_{S}∼\text{Gamma}\left(0,\;\tau_{S}\right)$$

are model priors. The model was fitted for *i* = 1, 2, … , *N* where *N* represents the total number of observations, and *k *=* *1, 2, … number of fixed effects. Here, *M* represents the movement metric examined for each observation *i*. *β*_Ref Before_ represents the intercept, that is, the Reference treatment prior to treatment implementation. *β*_Norab_ represents the effect of the No Rabbit Hunting treatment, and *β*_Red_ characterizes the effect of the Reduced treatment. *β*_Norab × After_ symbolizes the change in each examined movement metric after the implementation of the rabbit hunting treatment in the No Rabbit Hunting treatment. *β*_Red × After_ denotes the change in each examined movement metric after the implementation of the rabbit hunting treatment within the Reduced treatment. *X* represents a dummy variable for each respective fixed effect noted. The random effect of site denoted by *σ*_S_. Movement metrics such as step length that had multiple points throughout a movement track had the additional random effect of track signified by *σ*_T_. Models for distance to hardwood and scrub/shrub were modeled with a negative binomial distribution instead of a Gaussian distribution.

We used Markov chain Monte Carlo (MCMC) to estimate the posterior distributions of the model parameters. We generated three MCMC chains, a thinning rate of one, and varying iterations and burn‐in values depending on the trajectory metric being modeled. Iterations and burn‐in values used ensured an adequate number to characterize the posterior distributions that MCMC chains showed no indications of autocorrelation or effects of initial values and that all chains converged. We checked chain convergence using the Gelman–Rubin statistic, R‐hat, which compared between and within chain variation (Gelman, Carlin, Stern, & Rubin, 2004). R‐hat values below 1.1 indicate convergence. Values of all estimated parameters had an R‐hat value of 1.1 or below.

To estimate effects of the BACI study, we calculated posterior distributions for BACI ratios to determine the change in metrics in response to treatments while accounting for pretreatment differences. The BACI ratio was calculated using the ratio of pretreatment differences, *R*_t|ref Before_ = *M*_Treatment before_/*M*_Reference before_, where M is the posterior distribution for a respective metric and then doing the same with the after‐treatment differences, *R*_t|ref After_ = *M*_Treatment after_/*M*_Reference after_. We then estimated the posterior distribution of the treatment effect of each trajectory metric as R*_M_*_ BACI_ = R_t|ref After_/R_t|ref Before_. That is, for each MCMC sample, we calculated the mean ratio from the treatments for each trajectory metric after the rabbit hunting treatments were implemented and the mean ratio for the treatments for each trajectory metric before the rabbit hunting treatments were implemented and divided them. We estimated the mean, 2.5 and 97.5 percentiles for the distribution of each R*_M_*_ BACI_. BACI ratios deviating from 1 indicate an effect of the treatment implementation, with values >1 indicating a positive relationship and values <1 representing a negative relationship. Percent change is determined by subtracting 1 from the BACI ratio multiplied by 100 for positive effects or 1 minus the BACI ratio multiplied by 100 for negative effects. For example, a BACI ratio of 1.30 would result in a 30% increase in the tested metric, while a BACI ratio of 0.70 would result in a 30% decrease. Posterior overlap was used to denote the effect of a movement metric, which we here define as the amount of the posterior distribution that overlaps 1 for the BACI ratios.

## 3 RESULTS

### 3.1 Movement summary

Bobwhite coveys were naturally dispersed across the study site both years this, as well as the random selection of the treatment areas, resulted in the varying levels of coveys in each treatment (Table S1). We collected daily movement tracks from all 73 unique tagged coveys (35 before, 38 after) during the duration of the rabbit and bobwhite hunting seasons from November 2016 to February 2018, with an average of 23 locations per track. Movement tracks were collected on coveys during 18 bobwhite hunt days and 55 rabbit hunt days in total between both years. Summary of movement metrics examined is listed within Table S2. After the implementation of rabbit hunting treatments, 122 rabbit hunters were reported at Di‐Lane from hunter sign‐in sheets, with 35% of hunts occurring in the Reduced treatment and 64% occurring in the Reference treatment.

### 3.2 Hypotheses results

**Figure 2:**
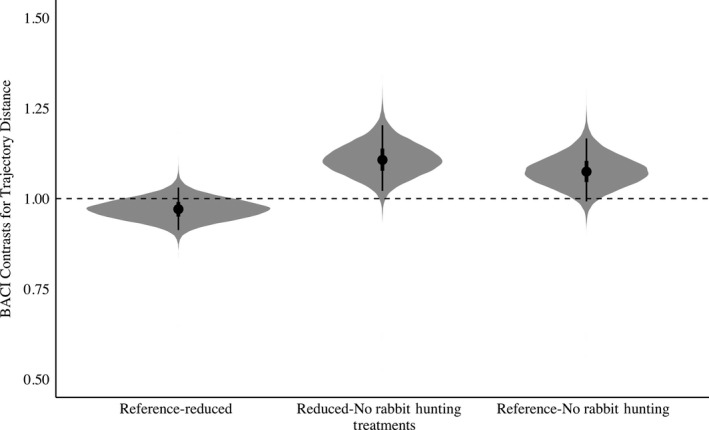
BACI contrasts with shaded regions representing the full posterior distribution for Northern Bobwhite (*Colinus virginianus*) trajectory distance in relation to rabbit hunting. Points indicate mean values for trajectory distance, with lines representing the 50% and 95% credible interval. BACI contrasts above 1 indicate an increase in trajectory distance while BACI contrasts below 1 indicate a decrease. Reference‐Reduced denotes the effect of reducing rabbit hunting from 5 to 3 days. Reduced‐No Rabbit Hunting denotes the effect of decreasing rabbit hunting from 3 to 0 days. Reference‐No Rabbit Hunting denotes the effect of decreasing rabbit hunting from 5 to 0 days

**Figure 3:**
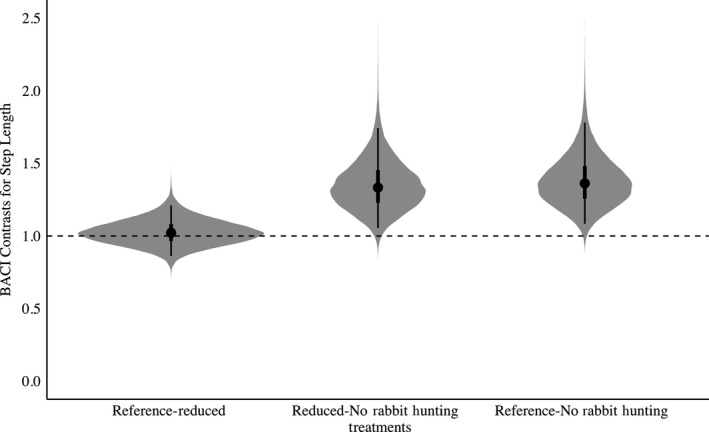
BACI contrasts with shaded regions representing the full posterior distribution for Northern Bobwhite (*Colinus virginianus*) step length in relation to rabbit hunting. Points indicate mean values for step length, with lines representing the 50% and 95% credible interval. BACI contrasts above 1 indicate an increase in step length while BACI contrasts below 1 indicate a decrease. Reference‐Reduced denotes the effect of reducing rabbit hunting from 5 to 3 days. Reduced‐No Rabbit Hunting denotes the effect of decreasing rabbit hunting from 3 to 0 days. Reference‐No Rabbit Hunting denotes the effect of decreasing rabbit hunting from 5 to 0 days

**Figure 4:**
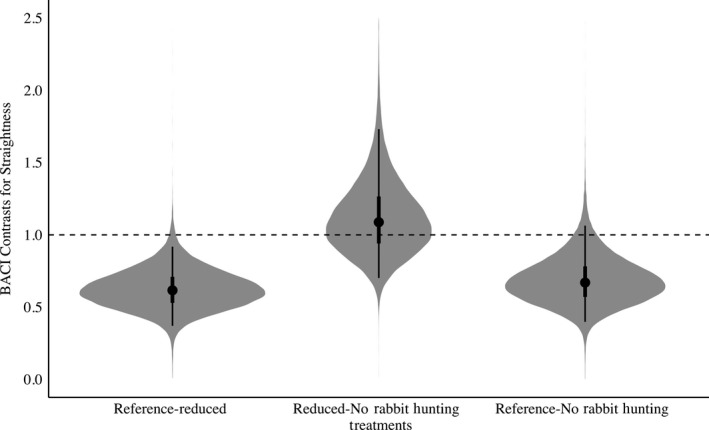
BACI contrasts with shaded regions representing full posterior distribution for Northern Bobwhite (*Colinus virginianus*) path straightness in relation to rabbit hunting. Points indicate mean values for path straightness, with lines representing the 50% and 95% credible interval. BACI contrasts above 1 indicate an increase in path straightness while BACI contrasts below 1 indicate a decrease. Reference‐Reduced denotes the effect of reducing rabbit hunting from 5 to 3 days. Reduced‐No Rabbit Hunting denotes the effect of decreasing rabbit hunting from 3 to 0 days. Reference‐No Rabbit Hunting denotes the effect of decreasing rabbit hunting from 5 to 0 days

**Figure 5:**
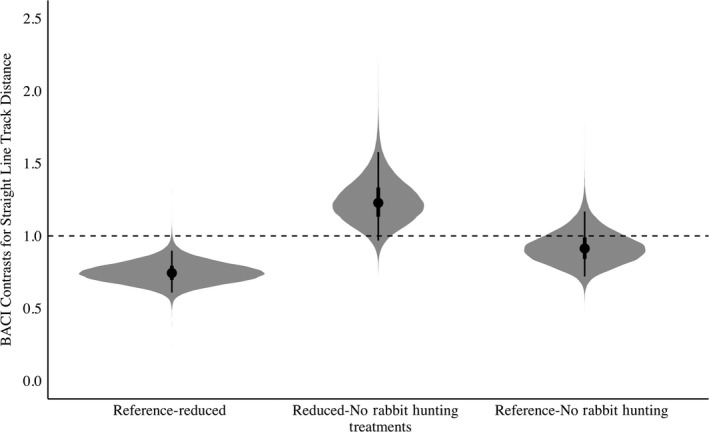
BACI contrasts with shaded regions representing the full posterior distribution for Northern Bobwhite (*Colinus virginianus*) straight‐line track distance in relation to rabbit hunting. Points indicate mean values for straight‐line track distance, with lines representing the 50% and 95% credible interval. BACI contrasts above 1 indicate an increase in straight‐line track distance while BACI contrasts below 1 indicate a decrease. Reference‐Reduced denotes the effect of reducing rabbit hunting from 5 to 3 days. Reduced‐No Rabbit Hunting denotes the effect of decreasing rabbit hunting from 3 to 0 days. Reference‐No Rabbit Hunting denotes the effect of decreasing rabbit hunting from 5 to 0 days

In general, there was support for the threat sensitivity hypothesis. We observed an overall decrease in bobwhite movement in the presence of rabbit hunting indicating partial support for the increased antipredator behavior threat sensitivity prediction (Figures 2 and 3). When rabbit hunting was reduced to 3 hunting days, trajectory distance, or overall daily movement, would decrease 3% when compared to the Reference treatment (Posterior Overlap = 0.16, Table S3, Figure 2). When compared to the Reference, when rabbit hunting was absent, bobwhite increased their trajectory distance by 8% (Posterior Overlap = 0.04, Table S3, Figure 2). Trajectory distance was also increased 11% when rabbit hunting was further reduced from 3 hunting days to 0 days (Posterior Overlap = 0.01, Table S3, Figure 2). Similarly, in the absence of rabbit hunting, bobwhite increased their step length by 38% when compared to the Reference (Posterior Overlap = 0.01, Table S3, Figure 3). Reducing rabbit hunting to 0 days from 3 days increased step length 33% (Posterior Overlap = 0.01, Table S3, Figure 3). Similarly, track straightness decreased 38% when rabbit hunting was reduced (Posterior Overlap = 0.01, Table S3, Figure 4). Track straightness decreased 32% when rabbit hunting decreased from the Reference to 0 days (Posterior Overlap = 0.04, Table S3, Figure 4). Furthermore, bobwhite would decrease their straight‐line track distance 25% when rabbit hunting was reduced to 3 days per week indicating a more tortuous path (Posterior Overlap = 0.01, Table S3, Figure 5).

**Figure 6:**
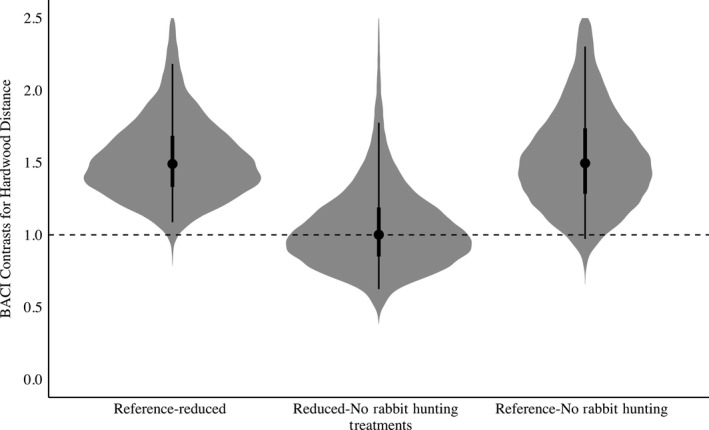
BACI contrasts with shaded regions representing the full posterior distribution for Northern Bobwhite (*Colinus virginianus*) distance from hardwood in relation to rabbit hunting. Points indicate mean values for distance from hardwood, with lines representing the 50% and 95% credible interval. BACI contrasts above 1 indicate an increase in distance from hardwood while BACI contrasts below 1 indicate a decrease. Reference‐Reduced denotes the effect of reducing rabbit hunting from 5 to 3 days. Reduced‐No Rabbit Hunting denotes the effect of decreasing rabbit hunting from 3 to 0 days. Reference‐No Rabbit Hunting denotes the effect of decreasing rabbit hunting from 5 to 0 days

**Figure 7:**
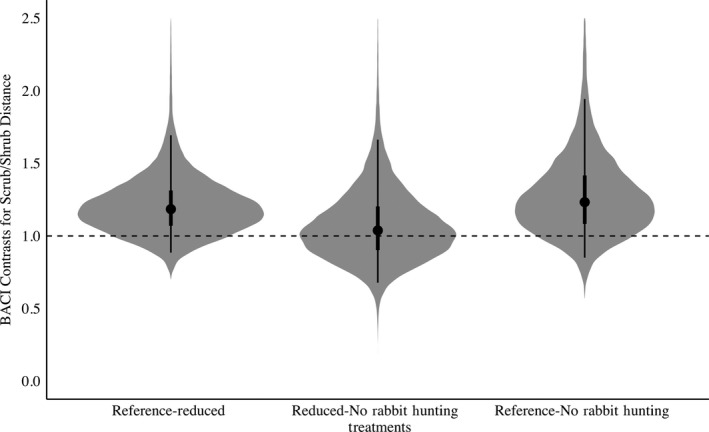
BACI contrasts with shaded regions representing the full posterior distribution for Northern Bobwhite (*Colinus virginianus*) distance from scrub/shrub in relation to rabbit hunting. Points indicate mean values for distance from scrub/shrub, with lines representing the 50% and 95% credible interval. BACI contrasts above 1 indicate an increase in distance from scrub/shrub while BACI contrasts below 1 indicate a decrease. Reference‐Reduced denotes the effect of reducing rabbit hunting from 5 to 3 days. Reduced‐No Rabbit Hunting denotes the effect of decreasing rabbit hunting from 3 to 0 days. Reference‐No Rabbit Hunting denotes the effect of decreasing rabbit hunting from 5 to 0 days

The risky space hypothesis was supported because bobwhite increased their distance from hardwoods and escape cover (scrub/shrubs) when rabbit hunting was absent when compared to the Reference treatment. Reducing rabbit hunting from 5 to 3 days yielded a 54% increase in bobwhite distance from hardwoods (Posterior Overlap = 0.01, Table S3, Figure 6). There was a 59% increase in distance from hardwoods when rabbit hunting was absent compared with the Reference (Posterior Overlap = 0.03, Table S3, Figure 6). There was no difference between the Reduced rabbit hunting and No Rabbit Hunting treatment (Posterior Overlap = 0.51). Relatedly, there was a 22% increase in distance from escape cover when rabbit hunting was reduced from 5 to 3 days (Posterior Overlap = 0.13, Table S3, Figure 7). Similarly, distance from scrub/shrub escape cover also increased when rabbit hunting was removed compared to the Reference, with a distance increase of 28% (Posterior Overlap = 0.14, Table S3, Figure 7) but no difference between 3 and 0 days (Posterior Overlap = 0.43). The Protean behavior was mostly unsupported. Contrary to its prediction, bobwhite increased the tortuosity of their paths by 32% (Posterior Overlap = 0.04, Table S3, Figure 4) when rabbit hunting risk was eliminated compared with the Reference.

## 4 DISCUSSION

We found general support for the threat sensitivity and risky space hypotheses whereas the Protean behavior hypothesis was mostly unsupported. Specifically, bobwhite increased movement, path tortuosity, and distance from escape cover in the absence of rabbit hunting. This increase in movement and distance from escape cover within areas with no rabbit hunting may indicate that bobwhites were able to properly perceive lower risk of predation within these treatments and behave accordingly. The use of the BACI design allowed us to reduce the possibility of confounding effects of site and annual variation while overcoming the constraints of implementing large‐scale manipulations such as cost and area restrictions.

Increased antipredator behavior by bobwhite was observed within treatments with rabbit hunting pressure through an overall reduction in movement, indicating partial support for the increased antipredator behavior threat sensitivity prediction stemming from the threat‐sensitive hypothesis. The observed decline in movement was similar between the Reference and Reduced treatments, indicating similar reactions to the presence of rabbit hunting regardless of the number of hunting days. The increased antipredator behavior observed can be described by a common bobwhite antipredator behavior known as holding, which allows individuals to remain cryptic within their landscape (Stoddard, 1931). The two most well‐known antipredator responses of bobwhite are holding and flushing behavior (Stoddard, 1931). The behavior used depends on the individual's perception of the threat. While holding behavior allows individuals to remain camouflaged in their environment, bobwhite tend to flush when they assess a high‐risk situation (McGrath et al., 2018). Flushing is reserved for immediate danger, as it is the most energetically costly and has the largest fitness cost to the individual by decreasing activities such as feeding and increasing visibility to predators (Ydenberg & Dill, 1986).

This evaluation is not necessarily a result of direct predator confrontation, but indirect landscape cues (Schmidt & Kuijper, 2015) such as the auditory cues of hunting dogs (Ciuti et al., 2012; Cromsigt et al., 2013). While both rabbit hunting and quail hunting involve the use of hunting dogs, rabbits are often hunted with hounds (Lowe, 1958), whereas quail are hunted with pointing dogs (McGrath et al., 2018). Many studies have found that loud noise can elicit antipredator behavior, as it is considered a threatening stimulus (Frid & Dill, 2002; Goldstein et al., 2005; McRoberts et al., 2011). In the case of our study, the use of hound dogs, who often howl, for rabbit hunting may provide bobwhite with an auditory cue pertaining to the level of risk on the landscape. This assessment of risk indicates support for the threat sensitivity hypothesis demonstrating that bobwhite are able to perceive an increase in hunting pressure within the respective areas and discern that while rabbit hunting is perceived as a threat, it does not require immediate energetically costly escape tactics.

Bobwhite did not show an increase in erratic movement, or Protean behavior, as a result of rabbit hunting pressure. Decreased path tortuosity within the Reference treatment may be a product of the specific predator present, as predator type determines antipredator escape tactics (Perkins, Boal, Rollins, & Perez, 2014). Recent literature has demonstrated an increase in bobwhite path tortuosity as a result of evading avian predators when compared to terrestrial threats, such as hunters (Perkins et al., 2014). Moreover, it has been suggested that Protean behavior is energetically costly and may only be utilized if prey are directly targeted by a predator (Jones et al., 2011). These findings reinforce our assessment that bobwhite use other strategies to mediate the risk of rabbit hunting instead of Protean behavior. Increased path tortuosity in the absence of rabbit hunting may be due to an increase in foraging behavior, which also results in tortuous paths, and not Protean behavior. McGrath et al. (2018) found that increased hunting pressure resulted in a decline in bobwhite foraging behavior. The decreased hunting pressure in the absence of rabbit hunting may explain the observed increase in path tortuosity within these areas, allowing bobwhite to decrease antipredator behavior and forage more freely. However, further examination of this subject is needed as current data do not allow us to discern between foraging and particular types of predator avoidance behavior that may be affecting path tortuosity.

Bobwhite were found to increase their distance from escape cover and hardwoods when rabbit hunting pressure decreased, indicating support for the risky space hypothesis. These findings suggest that bobwhite maintained closer proximity to hardwoods and scrub/shrub escape cover as a result of high rabbit hunting pressure. In general, prey has an increased perception of predation risk as their distance from refugia increases and as predator abundance increases (Stankowich & Blumstein, 2005). Perkins et al. (2014) found that bobwhite pursued by hunters tended to use greater cover and that the auditory threat stimulus of gunshots and hunting dogs may elicit this response. Therefore, the presence of rabbit hunters on the landscape may have prompted bobwhite to maintain closer proximity to refuge, even when not being directly pursued. Bobwhite used poorer habitat (e.g., hardwoods) to mediate the perceived higher relative risk on the landscape with the increase in rabbit hunting pressure. Increased use of poor habitat during high rabbit hunting pressure may be a result of a trait‐mediated effect (Cresswell, 2008). Trait‐mediated effects can have negative effects on individual fitness through reduced foraging time or forcing individuals from a profitable area due to predation risk (Cresswell, 2008). Being forced from profitable areas to poorer habitats, such as hardwoods, may also increase susceptibility to predation by other species. Kotler (1984) demonstrated that species of desert rodents least vulnerable to predators tended to forage in open areas, whereas the most vulnerable species were restricted to covered areas. Individuals who tend to avoid areas of high‐risk exposure to a predator would then spend their lives in energetically poor habitats (Abrahams & Dill, 1989). In the case of a species with a short life expectancy, such as bobwhite, this is not a suitable option. Therefore, when individuals utilize these poor habitats, they may be more likely to engage in uncertain or risk‐sensitive foraging behavior to ensure they receive the necessary nutrients for survival (Abrahams & Dill, 1989). The increase in distance from refuge observed within the Reduced and No Rabbit Hunting treatments would therefore be an effect of reduced hunting pressure on the landscape, resulting in less restricted movement.

Taken collectively our results indicate that bobwhite adjust their movements to the spatial patterns of rabbit hunters to mediate the perceived predation risk. Bobwhite decreased overall movement in areas in which rabbit hunting occurred regardless of the number of hunting days. Nevertheless, the full extent of the effect of rabbit hunting on bobwhite still needs to be investigated. Heightened antipredator behavior through decreased movement may assist with bobwhite predator avoidance by taking advantage of their cryptic coloration in the landscape (Stoddard, 1931). However, decreased movement and increased use of poor habitats such as hardwoods may also have negative effects as a result of reduced foraging time or increased susceptibility to other predators. Additionally, heightened use of antipredator behavior in avian species has been shown to decrease fitness through a decline in clutch survival and fecundity (Dudeck, Clinchy, Allen, & Zanette, 2018; Zanette et al., 2011). Future research should attempt to quantify the effect of decreased movement on bobwhite fitness through the evaluation of foraging time and survival. Research should also focus on the physiological effects of rabbit hunting on bobwhite through the examination of stress hormones to help assess the effects of rabbit hunting on bobwhite fitness and subsequent population dynamics.

## CONFLICT OF INTEREST

None declared.

## AUTHOR CONTRIBUTIONS

JM, IP, and JM conceived the ideas and designed methodology; JM, NW, and RG collected the data; JM and JM analyzed the data; JM and JM led the writing of the manuscript. All authors contributed critically to the drafts and gave final approval for publication.

## DATA AVAILABILITY

Data were archived using Dryad, https://doi.org/10.5061/dryad.b3k21db.
