# Supplementary material for: Nonconsumptive effects of hunting on a nontarget game bird
Source: Ecol Evol. 2019 Jul 30;9(16):9324–33. doi: 10.1002/ece3.5479 (PMC6706207; doi:10.1002/ece3.5479)
Supplement: Supplementary file 1 [file ECE3-9-9324-s001.docx]

Table S1. Northern Bobwhite (*Colinus virginianus*) movement track distribution on Di-Lane WMA. Time period Before references the time prior rabbit hunting treatment implementation during 2016-2017. After denotes the time period after the implementation of rabbit hunting treatments during 2017-2018. Reference is the rabbit hunting treatment in which hunting may occur 5 days per week, Reduced is where rabbit hunting may occur 3 days per week, and No Rabbit Hunting allows for 0 days per week. Bobwhite coveys were naturally dispersed across the study site both years, this, as well as the random selection of the treatment areas, resulted in the varying levels of coveys in each treatment.

|  | Time Period | |
| --- | --- | --- |
| Treatment | Before | After |
| Reference | 14 | 17 |
| Reduced | 16 | 13 |
| No Rabbit Hunting | 5 | 8 |
