# Supplementary material for: Nonconsumptive effects of hunting on a nontarget game bird
Source: Ecol Evol. 2019 Jul 30;9(16):9324–33. doi: 10.1002/ece3.5479 (PMC6706207; doi:10.1002/ece3.5479)
Supplement: Supplementary file 2 [file ECE3-9-9324-s002.docx]

Table S2. Summary of Northern Bobwhite (*Colinus virginianus*) movement metrics examined before and after the implementation of rabbit hunting treatments. Reference is the rabbit hunting treatment in which hunting may occur 5 days per week, Reduced is where rabbit hunting may occur 3 days per week, and No Rabbit Hunting allows for 0 days per week.

| Movement metric | Treatment | Period | Mean | SD |
| --- | --- | --- | --- | --- |
| Trajectory distance | Reference | Before | 1283.60 | 448.35 |
|  |  | After | 1025.10 | 369.56 |
|  | Reduced | Before | 1309.70 | 620.04 |
|  |  | After | 871.40 | 325.24 |
|  | No Rabbit Hunting | Before | 694.50 | 250.12 |
|  |  | After | 889.90 | 303.96 |
| Step-length | Reference | Before | 51.34 | 39.02 |
|  |  | After | 45.49 | 39.20 |
|  | Reduced | Before | 55.44 | 38.05 |
|  |  | After | 39.91 | 34.20 |
|  | No Rabbit Hunting | Before | 33.39 | 33.32 |
|  |  | After | 42.87 | 42.55 |
| Straightness | Reference | Before | 0.10 | 0.08 |
|  |  | After | 0.23 | 0.16 |
|  | Reduced | Before | 0.18 | 0.11 |
|  |  | After | 0.18 | 0.17 |
|  | No Rabbit Hunting | Before | 0.28 | 0.18 |
|  |  | After | 0.22 | 0.10 |
| Straight-line track distance | Reference | Before | 103.69 | 91.46 |
|  |  | After | 182.73 | 154.13 |
|  | Reduced | Before | 177.85 | 90.50 |
|  |  | After | 101.69 | 77.64 |
|  | No Rabbit Hunting | Before | 167.11 | 142.19 |
|  |  | After | 166.31 | 96.51 |
| Hardwood Distance | Reference | Before | 77.37 | 62.76 |
|  |  | After | 49.05 | 47.82 |
|  | Reduced | Before | 82.09 | 131.20 |
|  |  | After | 139.36 | 116.98 |
|  | No Rabbit Hunting | Before | 77.03 | 116.34 |
|  |  | After | 90.75 | 60.06 |
| Scrub/Shrub Distance | Reference | Before | 22.33 | 20.85 |
|  |  | After | 41.75 | 43.69 |
|  | Reduced | Before | 15.73 | 19.54 |
|  |  | After | 25.53 | 27.85 |
|  | No Rabbit Hunting | Before | 18.31 | 19.25 |
|  |  | After | 20.62 | 17.63 |
