# Supplementary material for: Nonconsumptive effects of hunting on a nontarget game bird
Source: Ecol Evol. 2019 Jul 30;9(16):9324–33. doi: 10.1002/ece3.5479 (PMC6706207; doi:10.1002/ece3.5479)
Supplement: Supplementary file 3 [file ECE3-9-9324-s003.docx]

Table S3. Mean BACI ratio and credible intervals for Northern Bobwhite (*Colinus virginianus*) movement metrics in relation to three different levels of rabbit hunting. BACI ratios above 1 indicate a positive relationship while BACI ratios below 1 indicate a indicate a negative. Reduced-No Rabbit Hunting denotes the effect of decreasing rabbit hunting from 3 days to 0 days. Reference-Reduced denotes the effect of reducing rabbit hunting from 5 days to 3 days. Reference-No Rabbit Hunting denotes the effect of decreasing rabbit hunting from 5 days to 0 days.

| Movement metric | BACI-ratio | 0.025 | 0.250 | Mean | 0.750 | 0.975 | Posterior Overlap |
| --- | --- | --- | --- | --- | --- | --- | --- |
| Trajectory distance | Reference-No Rabbit Hunting | 0.99 | 1.05 | 1.08 | 1.10 | 1.17 | 0.04 |
|  | Reference-Reduced | 0.91 | 0.95 | 0.97 | 0.99 | 1.03 | 0.16 |
|  | Reduced-No Rabbit Hunting | 1.02 | 1.07 | 1.11 | 1.13 | 1.20 | 0.01 |
| Step-length | Reference-No Rabbit Hunting | 1.09 | 1.25 | 1.38 | 1.48 | 1.78 | 0.01 |
|  | Reference-Reduced | 0.86 | 0.96 | 1.03 | 1.08 | 1.20 | 0.40 |
|  | Reduced-No Rabbit Hunting | 1.06 | 1.23 | 1.33 | 1.47 | 1.75 | 0.01 |
| Straightness | Reference-No Rabbit Hunting | 0.36 | 0.57 | 0.68 | 0.79 | 1.07 | 0.04 |
|  | Reference-Reduced | 0.32 | 0.53 | 0.62 | 0.72 | 0.93 | 0.01 |
|  | Reduced-No Rabbit Hunting | 0.68 | 0.93 | 1.05 | 1.27 | 1.82 | 0.36 |
| Straight-line track distance | Reference-No Rabbit Hunting | 0.71 | 0.84 | 0.92 | 0.99 | 1.17 | 0.23 |
|  | Reference-Reduced | 0.59 | 0.69 | 0.75 | 0.80 | 0.90 | 0.01 |
|  | Reduced-No Rabbit Hunting | 0.96 | 1.13 | 1.22 | 1.33 | 1.58 | 0.05 |
| Hardwood distance | Reference-No Rabbit Hunting | 0.98 | 1.29 | 1.59 | 1.78 | 2.70 | 0.03 |
|  | Reference-Reduced | 1.09 | 1.33 | 1.54 | 1.69 | 2.28 | 0.01 |
|  | Reduced-No Rabbit Hunting | 0.63 | 0.84 | 0.98 | 1.19 | 1.82 | 0.51 |
| Scrub/Shrub distance | Reference-No Rabbit Hunting | 0.85 | 1.08 | 1.28 | 1.42 | 2.00 | 0.14 |
|  | Reference-Reduced | 0.88 | 1.07 | 1.22 | 1.31 | 1.72 | 0.13 |
|  | Reduced-No Rabbit Hunting | 0.67 | 0.90 | 1.02 | 1.20 | 1.67 | 0.43 |
